# Microbiome dysbiosis in patients with chronic endometritis and *Clostridium tyrobutyricum* ameliorates chronic endometritis in mice

**DOI:** 10.1038/s41598-024-63382-4

**Published:** 2024-05-30

**Authors:** Jiujiu Liu, Xiaorong Tang, Lei Chen, Yue Zhang, Jinfang Gao, Aiming Wang

**Affiliations:** 1 The Second School of Clinical Medicine, Southern Medical University, Guangzhou, 510000 China; 2 Department of Obstetrics and Gynecology, The Sixth Medical Center of PLA General Hospital, No. 6 Fucheng Road, Haidian District, Beijing, 100000 China; 3 Department of Obstetrics and Gynecology, Jiyuan People’s Hospital, Jiyuan, 454650 China

**Keywords:** Abundance, *Clostridium tyrobutyricum*, Diagnosis, Diversity, Endometritis, Microbiota, TLR4/NF-κB pathway, Microbiology, Microbial communities

## Abstract

Chronic endometritis is associated with the imbalance of female reproductive tract microbiota and pathogenic microbial infection. This study aimed to identify the specific changes in the endometrial microbiome in patients with endometritis and to explore how *Clostridium tyrobutyricum* (*C.t*) influences the progression of endometritis in mice for further elucidating endometritis pathogenesis. For this purpose, endometrial tissues from 100 participants were collected and divided into positive, weakly positive, and negative groups based on CD138 levels, while endometrial microbiome differences were detected and analyzed using 16S rRNA gene sequencing. *Staphylococcus aureus* (*S. aureus*)-induced endometritis mouse model was established, followed by treatment with *C.t*, and inflammatory response, epithelial barrier, and TLR4/NF-κB pathway were evaluated. Results showed that α- and β-diversity was significantly lower in the positive group compared with the weakly positive or negative groups, where the negative group had more unique operational taxonomic units. The abundance of *Proteobacteria* was found to be increased, while that of *Actinobacteria*, *Firmicutes,* and *Bacteroidetes* was found to be reduced in the positive group, while the area under the curve value was found to be 0.664. Furthermore, *C.t* treatment resulted in the alleviation of *S. aureus*-induced inflammatory response, epithelial barrier damage, and activation of the TLR4/NF-κB pathway in mice. Clinical samples analysis revealed that the diversity and abundance of microbiota were altered in patients with endometritis having positive CD138 levels, while mechanistic investigations revealed *C.t* alleviated *S. aureus*-induced endometritis by inactivating TLR4/NF-κB pathway. The findings of this study are envisaged to provide a diagnostic and therapeutic potential of microbiota in endometritis.

## Introduction

Chronic endometritis is an inflammatory condition affecting the endometrium structure with multiple etiologies and may remain asymptomatic^1^. Untreated endometritis has been found to result in pus accumulation in the uterine cavity and pelvic inflammation, ultimately translating into infertility^2^. Statistically, endometritis was reported to occur in 7–56% of individuals experiencing recurrent spontaneous abortion and in 7.7–44% of those facing repeated embryo implantation failures^3^. Antibiotic treatment is considered a preferred treatment strategy for endometritis based on clinically available options^4^ but is associated with demerits of non-specific, residual, microbial imbalance and the subsequent antibiotic resistance^5,6^, in addition to being a primary reason for the increased risk of pregnancy in patients with endometritis^7^. Hence, it is imperative to elucidate endometritis pathogenesis and develop alternative safe and efficient treatment approaches in lieu of antibiotics.

Diverse microbiotas are intricately linked to the host, playing an important regulatory role in the host’s health and disease^8,9^. Microbes help maintain a dynamic balance in the body in a healthy state, but an imbalanced microbiome can translate into the onset and development of multiple diseases, including cancer, cardiovascular, and respiratory system diseases^10^. Similarly, an imbalanced reproductive tract microbiota has been reported to contribute to the onset and progression of endometritis^11^, where a significantly higher abundance of *Klebsiella*, *Lachnoclostridium_5*, and *Citrobacter* were found in mice with acute endometritis compared with controls^12^. Similar findings were also reported in patients with chronic endometritis, where the abundance of *Lactobacillus* was found to be reduced, while that of *Pseudomonas* and *Cutibacterium* were found to be elevated^13^. Moreover, significant differences were reported in the microbiome composition between endometritis and non-endometritis patients^14^. It is thus imperative to understand the alterations and regulatory mechanisms of microbiota in endometritis for developing novel therapeutic strategies.

Various kinds of microorganisms are inducers of chronic endometritis, such as *Enterobacteriaceae*, *Enterococcus*, *Streptococcus*, and *Staphylococcus*^15^. However, several microorganisms server the role as beneficial bacteria. *Clostridium tyrobutyricum* (*C.t*) is a Gram-positive anaerobic bacterium that produces acetic and butyric acid from glucose^16^. Butyric acid has been demonstrated to inhibit inflammation injury in previous studies^17,18^. *C.t* has probiotic properties, which can prevent the development of inflammatory bowel disease by changing gut microbiota composition^19^. It contributes to maintaining intestinal homeostasis by increasing regulatory T cells in the duodenum and T helper cell 17 in the ileum, thereby attenuating intestinal inflammation. In addition, Xiao et al.^20^ have reported that *C.t* can improve epithelial barrier dysfunction, and thus protect the intestines from LPS damage. Moreover, *C.t* reduces inflammation and maintain intestinal integrity and permeability by inhibiting apoptosis of epithelial cells^21^. Thus, the mechanism of *C.t* alleviating inflammation is multifaceted and very complex. Recent evidence has reported that *C.t* may decelerate endometritis progression based its anti-inflammation effect via inhibiting the release of pro-inflammatory cytokines and enhancing uterus barrier integrity^22^. Therefore, it is necessary to elucidate the role and the molecular mechanism of *C.t* in chronic endometritis.

This study utilized 16S rRNA gene sequencing to investigate changes in the microbiota of endometritis patients, in addition to exploring the effect of *Clostridium tyrobutyricum* (*C.t*) on the progression of endometritis and its molecular mechanism in *S. aureus* induced endometritis mouse model. The findings of this study are envisaged to provide a theoretical backing for the diagnosis and treatment of endometritis.

## Materials and methods

### Study design

**Figure 1:**
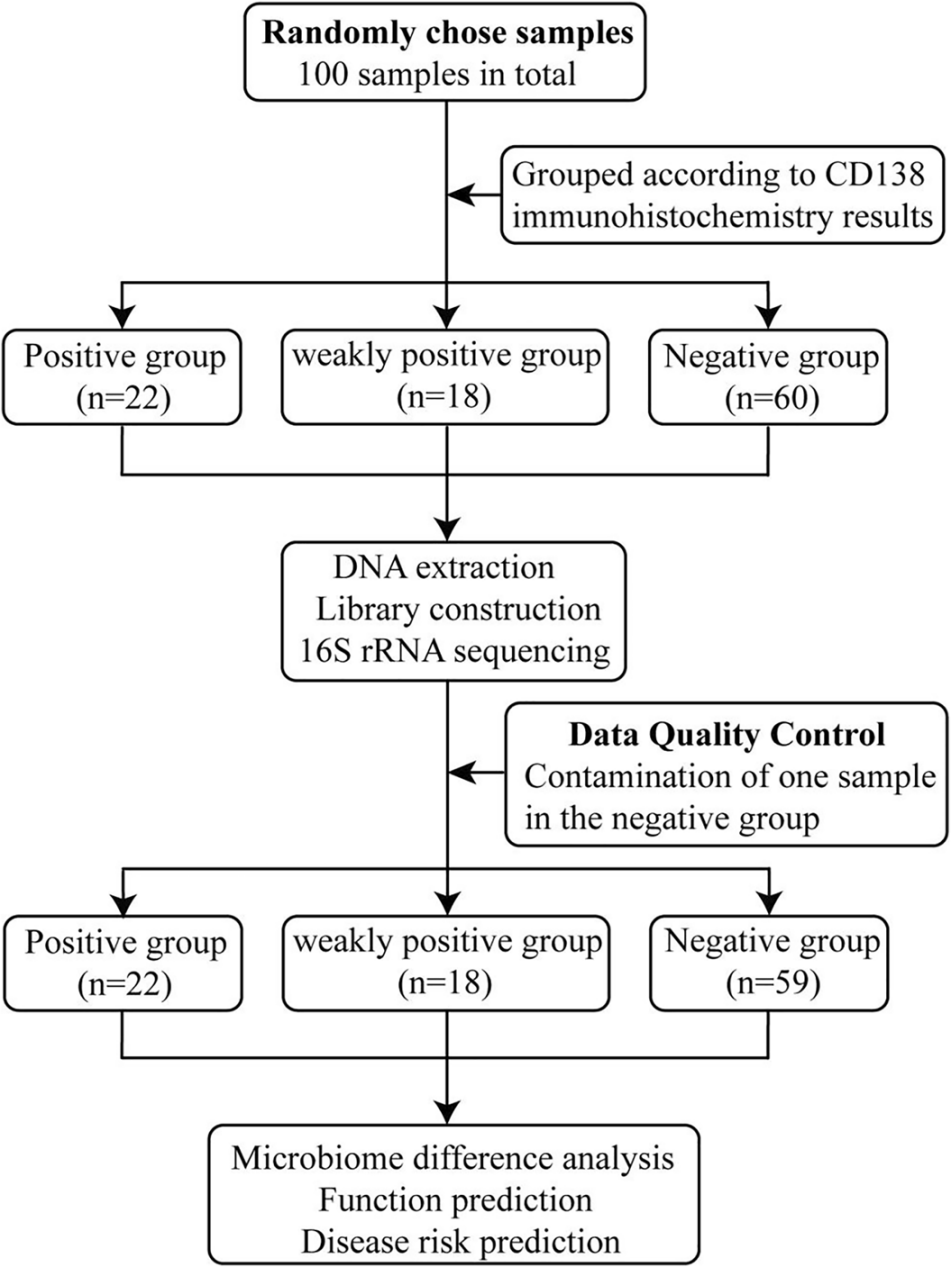
Experimental design flowchart.

A total of 100 participants were enrolled in this study. Endometrial tissues were collected from each participant. The participants were divided into three groups including positive (n = 22), weakly positive (n = 18), and negative (n = 60) groups according to the levels of CD138 in endometrial tissue using immunohistochemical staining. The tissues were analyzed using 16S rRNA gene sequencing. Contaminated samples were excluded. This study was approved by the Ethics Association of the Sixth Medical Center of PLA General Hospital (Approval No. HZKY2022-45), and all participants signed informed consent. The study design flowchart is shown in Fig. 1. The clinical characteristics of the participants in this study are shown in Table 1.

**Table 1:** Clinical characteristics of the study population.

Variable (mean ± SD)	Positive group (n = 22)	Weakly positive group (n = 18)	Negative group (n = 60)	pairwise comparison
Age (years)	31.55 ± 4.50	31.11 ± 2.14	32.03 ± 2.88	ns
BMI (kg/m^2^)	24.73 ± 1.28	24.44 ± 1.74	24.86 ± 2.32	ns
FSH (mIU/ml)	7.90 ± 1.96	8.27 ± 3.17	8.58 ± 3.03	ns
LH (mIU/ml)	5.29 ± 1.85	5.37 ± 2.29	5.55 ± 2.15	ns
FSH/LH ratio	1.73 ± 0.84	1.87 ± 1.07	1.81 ± 1.08	ns

*SD* standard deviation, *FSH* follicle-stimulating hormone, *LH* luteinizing hormone, *ns* no significant difference.

Participant inclusion criteria were: aged 25–40 years old; married; without contraception; regular sexual life for more than one year; infertility due to male and fallopian tube factors; in vitro fertilization failure at least one time. Participant exclusion criteria were: patients with other uterine lesions; abnormal results of the hormone tests; patients with vaginitis; patients with pelvic tenderness; hematological system diseases; and patients who have used antibiotics within the last one month.

### Collection of endometrial microorganisms and DNA extraction

Total DNA was extracted from endometrial specimens using the TIANamp Genomic DNA kit (TIANGEN, Beijing, China). DNA concentration and purity were determined using a NanodropTM 2000 ultramicrotome spectrophotometer (Thermo Scientific, Waltham, MA, USA). Finally, DNA was diluted to 1 ng/μL with sterile water according to the concentration and stored for further use.

## Polymerase chain reaction (PCR) amplification

The V3-V4 region of the 16S rRNA gene of the above DNA was amplified by PCR. The PCR reaction (20 μL reaction system) was performed using an ABI GeneAMP^®^ 9700 system. The specific primer sequences were shown below: forward 5′-CCTACGGGNGGCWGCAG-3′ and reverse 5′-GGACTACHVGGGTWTCTAAT-3′. The specific reaction conditions were as follows: 95 °C for 3 min (pre-denaturation); denaturation at 95 °C for 30 s; annealing at 55 °C for 30 s; extension at 72 °C for 45 s, total 30 cycles, and end extension at 72 °C for 8 min. After detecting the integrity and concentration of the PCR products, they were purified using the TIANgel purification kit (TIANGEN). The purified product was used to prepare the Illumina DNA library.

### Library construction and sequencing

Sequencing libraries were constructed using the Nextera XT DNA sample preparation kit (Illumina, Albany, NY, USA). The libraries were quantified using Qubit and quantitative real-time PCR, and sequencing was performed using the Illumina NovaSeq 6000 sequencing platform.

### Bioinformatics analysis

Microbiome analysis was performed using the QIIME2 software. The original data were importing the software, and quality filtering was analyzed using the q2-demux plug-in, followed by DADA2 algorithm for denoising, merging, and chimeric filtering. Thus, the calid data were acquired. Operational taxonomic units (OTUs) were picked using pick_de_novo_otus.py. The same OTUs were defined as the sequence similarity > 97%. Each OUT is represented by a representative sequence for species annotation and abundance analysis.

### Species annotation and bacterial diversity analysis

Species annotation of the obtained data according to the Silva database was conducted using the QIIME2 software. The rarefaction curve was plotted to view the microbial richness in samples. Additionally, α- and β- diversity was analyzed using the QIIME2 software. The of microorganisms α-diversity was assessed by detecting Chao1, Shannon, Simpson, and ACE indices. Meanwhile, β-diversity analysis was used to evaluate the differences in species complexity of the samples using principal-coordinate analysis (PCoA), non-metric multi-dimensional scaling (NMDS), and Anosim analysis. The bacteria were classified, analyzed, and compared at the levels of phylum, order, family, and genus. Subsequently, the endometrial microbiome was screened using linear discriminant analysis effect size (LEfSe) and linear discriminant analysis (LDA).

### Function prediction

Prokaryotic whole genome 16S rRNA gene sequences were obtained from the KEGG (Kyoto Encyclopedia of Genes and Genomes) database using Tax4Fun (an R package based on the 16S Silva database). These data were compared with the Silva database to mark Silva database functions. Our sequencing samples were clustered with the Silva database sequences as reference sequences to derive OTUs for functional annotation.

### Disease risk prediction

A mathematical model was generated according to the data of the sample, and model training was performed using random forest regression. The diagnostic value of the selected microorganisms was determined using the receiver operating characteristic (ROC) curve.

### Animal study

The animal study was approved by MDKN Biotechnology Co., Lt. Female BALB/c mice (20–25 g, 6–8-week-old) were purchased from Vital River (Beijing, China). All mice were kept in specific pathogen-free conditions with 12/12 h light/dark cycles, temperature of 24 ± 1 °C, and humidity of 50–70%. The mice were fed a normal standard diet (Trophic Animal Feed High-tech Co. Ltd, Nantong, China). All mice were allowed free access to food and water. All mice were divided into three groups after one-week acclimatization: control, *S. aureus*, and *S. aureus* + *C.t* groups, with 6 mice per group.

*S. aureus* (BNCC186335) and *C.t* (BNCC336696) were purchased from BeNa Culture Collection (Beijing, China). *S. aureus* was cultured in Mueller–Hinton broth (Hopebio, Qingdao, China) at 37 °C until the optical density (OD) value at 600 nm reached 2.0. *C.t* was cultured in Reinforced clostridial growth medium (Hopebio) at 37 °C under anaerobic environments. Then, the *S. aureus* and *C.t* were centrifuged at 5000*g* for 10 min and resuspended in sterile PBS. To establish the endometritis mouse model, *S. aureus* (1 × 10^7^ CFU/100 μL PBS) was injected into the bilateral uterine horn of the mice as previously described^23^. The mice in the control group were injected with 100 μL PBS into each bone of mice. In the *S. aureus* + *C.t* group, mice were given *C.t* (1 × 10^7^ CFU/200 μL PBS) by intragastric administration for one week (once per day), and then the endometritis model was generated. After the model was established for 24 h, the peripheral blood and uteri were collected from each mouse.

### Hematoxylin and eosin (H&E) staining assay

The uteri were fixed in 4% paraformaldehyde, and the histopathology was performed using H&E staining. The uterine tissues were embedded in paraffin and cut into sections at 4 μm of thickness. After dewaxing and rehydrating, the sections were stained with hematoxylin and eosin. The results were viewed using a light microscope and scored as previously described^24^.

### Determination of myeloperoxidase (MPO) activity

MPO activity was detected using an MPO assay kit (Jiancheng, Nanjing, China). The uterine tissues were homogenized to prepare 5% tissue homogenate. Then, the samples were incubated with reagents in the kit according to the manufacturer’s instructions. The absorbance was measured at 460 nm using an ultraviolet spectrophotometer.

### Enzyme-linked immunosorbent assay (ELISA)

The serum was isolated from peripheral blood by centrifuging at 1000*g* for 10 min. The levels of TNF-α and IL-1β were detected in the serum of mice using the mouse TNF-α ELISA kit and mouse IL-1β ELISA kit (both purchased from Elabscience, Wuhan, China), respectively.

### Western blot

The uterine tissues were lysed using radio immunoprecipitation assay lysis buffer. The isolated protein concentration was measured using a BCA protein colorimetric assay kit (Elabscience). Subsequently, the proteins were separated using 10% SDS-PAGE and then transferred onto polyvinylidene fluoride membranes. The membranes were incubated with primary antibodies at 4 °C for one night, and incubated with a secondary antibody at room temperature for 1 h. The protein bands were visualized using an excellent chemiluminescent substrate (ECL) detection kit (Elabscience).

The primary antibodies (Abcam, Cambridge, UK) were anti-ZO-1 (ab307799), anti-occludin (ab216327), anti-claudin-3 (ab214487), anti-TLR4 (ab13556), anti-p65 (ab32536), anti-phosphorylated (p)-p65 (ab76302)), anti-IKBα (ab32518), and anti-p-IKBα (ab133462). The secondary antibody was goat anti-rabbit IgG (ab6721; Abcam).

### Statistical analysis

Data were analyzed using the SPSS software (version 25.0; IBM, Armonk, NY, USA). The continuous variable data were analyzed using t-test and one-way ANOVA. The categorical variable data were analyzed using the Pearson chi-square test. *P* < 0.05 was determined to be statistically significant.

### Ethics approval and consent to participate

This study was approved by the Ethics Association of the Sixth Medical Center of PLA General Hospital (Approval No. HZKY2022-45), and all participants signed informed consent. The animal study was approved by MDKN Biotechnology Co., Lt. All experiments were performed in accordance with relevant guidelines and regulations.

## Results

### Patients overview

Endometrial tissue samples were collected from a total of 100 participants and divided into positive (n = 22), weakly positive (n = 18), and negative (n = 60) groups based on endometrial tissue CD138 levels. Moreover, the microbiota was analyzed using 16S rRNA sequencing, but due to contamination, the final negative group included 59 cases, followed by analyzing microbiome differences, functions, and disease risk prediction (Fig. 1). The clinical characteristics of participants in each group were compared. The results showed there were no significant differentiation of age, BMI, follicle-stimulating hormone (FSH), luteinizing hormone (LH), and FSH/LH ratio among these three groups (Table 1).

### Data quality control and endometrial microbial diversity

**Figure 2:**
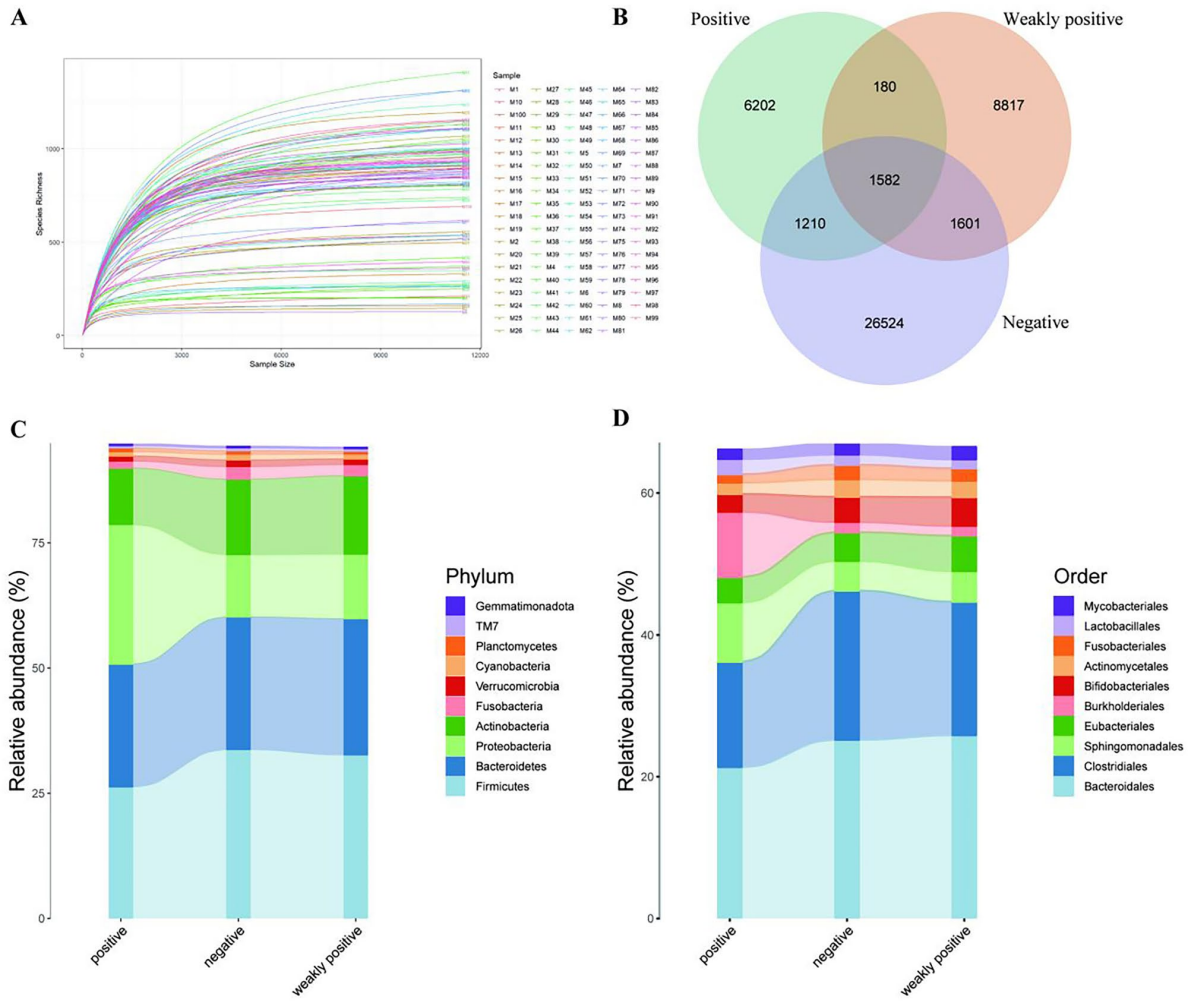
Differences in microbiome quality control and composition among different subgroups. (**A**) The rarefaction curve was smooth and close to saturation, indicating good species homogeneity. (**B**) The Venn diagram displaying the overlap between groups showed 1582 OTUs shared among groups, 6202 unique to the positive group (green), 8817 unique to the weakly positive group (orange), and 26,524 unique to the negative group (blue). (**C**) Alluvial plots showing the top 10 abundant microorganisms at the phylum level among different subgroups. (**D**) Alluvial plots showing the top 10 abundant microorganisms at the order level among different subgroups. OTUs, operational taxonomic units.

The microbes in each sample were identified using 16S rRNA sequencing, and the results of the rarefaction curve showed a smooth curve (Fig. 2A), suggesting a good homogeneity of the species with sufficient sequencing depth. The Venn diagram revealed that 1582 OTUs were similar in all groups, while 26,524, 6202, and 8817 OTUs were unique in the negative, positive, and weakly positive groups, respectively (Fig. 2B). These results indicated that the number of OTUs was higher in the negative group than in the positive and weakly positive groups; however, the difference between the positive and weakly positive groups was insignificant. Countering the top 10 abundant microbes in different subgroups at the phylum and order levels, plotting the alluvial plot (Fig. 2C) showed that *Firmicutes*, *Bacteroidetes*, *Proteobacteria*, and *Actinobacteria* accounted for > 75% in different groups at the phylum level, with insignificant difference between the weakly positive and negative groups. Moreover, the percentage of *Proteobacteria* was found to be increased, while *Actinobacteria* was decreased in the positive group, compared with the other two groups. Similarly, at the order level, *Bacteroidales*, *Eubacteriales*, *Sphingomonadales*, and *Burkholderiales* accounted for > 50% of the different subgroups, where compared with the weakly positive or negative group, the percentage of *Burkholderiales* in the positive group was found higher, whereas of *Eubacteriales* was reduced in the positive group (Fig. 2D).

### Endometrial microbial diversity

**Figure 3:**
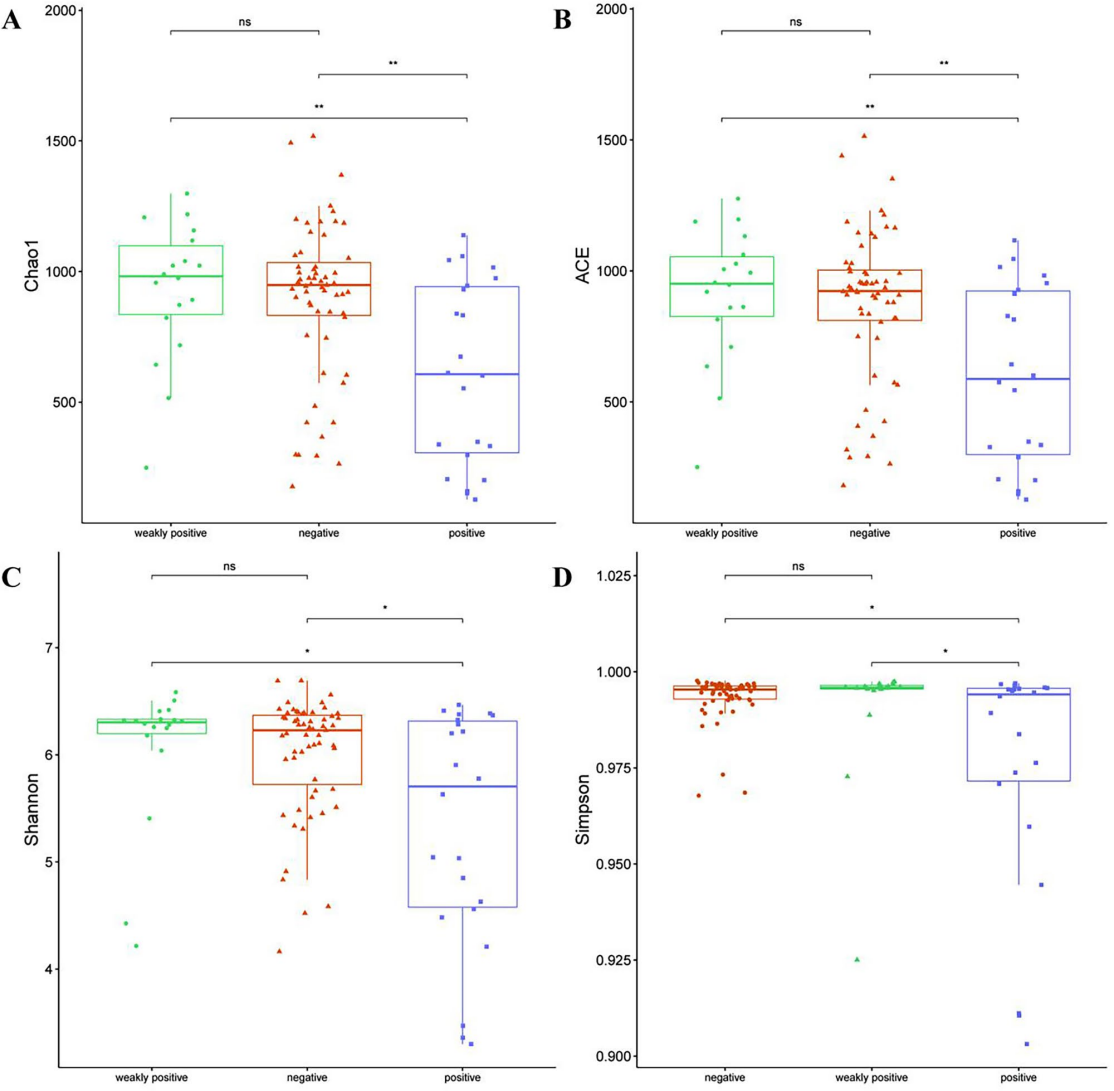
Differences in α-diversity index between the positive, negative, and weakly positive groups. (**A**, **B**) The Chao1 and ACE indices showed significantly lower microflora richness in the positive group than that in the weakly positive/negative group. (**C**, **D**) The Shannon and Simpson indices showed significant differences in microbial diversity between the positive group and the weakly positive/negative group. **P < 0.01. *P < 0.05. *ns* no significant.

The α- and β- diversity was examined to show the number, abundance, and evenness of the species, in addition to sample richness and homogeneity assessment by detecting the α-diversity index via Chao1, ACE, Shannon, and Simpson. Results showed that all these four indicators were significantly reduced in the positive group, compared with the weakly positive or negative group (Chao1 and ACE, *P* < 0.01; Shannon and Simpson, *P* < 0.01), but the difference between the weakly positive and negative groups was insignificant (Fig. 3A–D). These results implied that microbial richness and diversity were reduced in endometritis patients, suggesting that endometritis can induce an imbalance in the composition of the endometrial microbiota.

**Figure 4:**
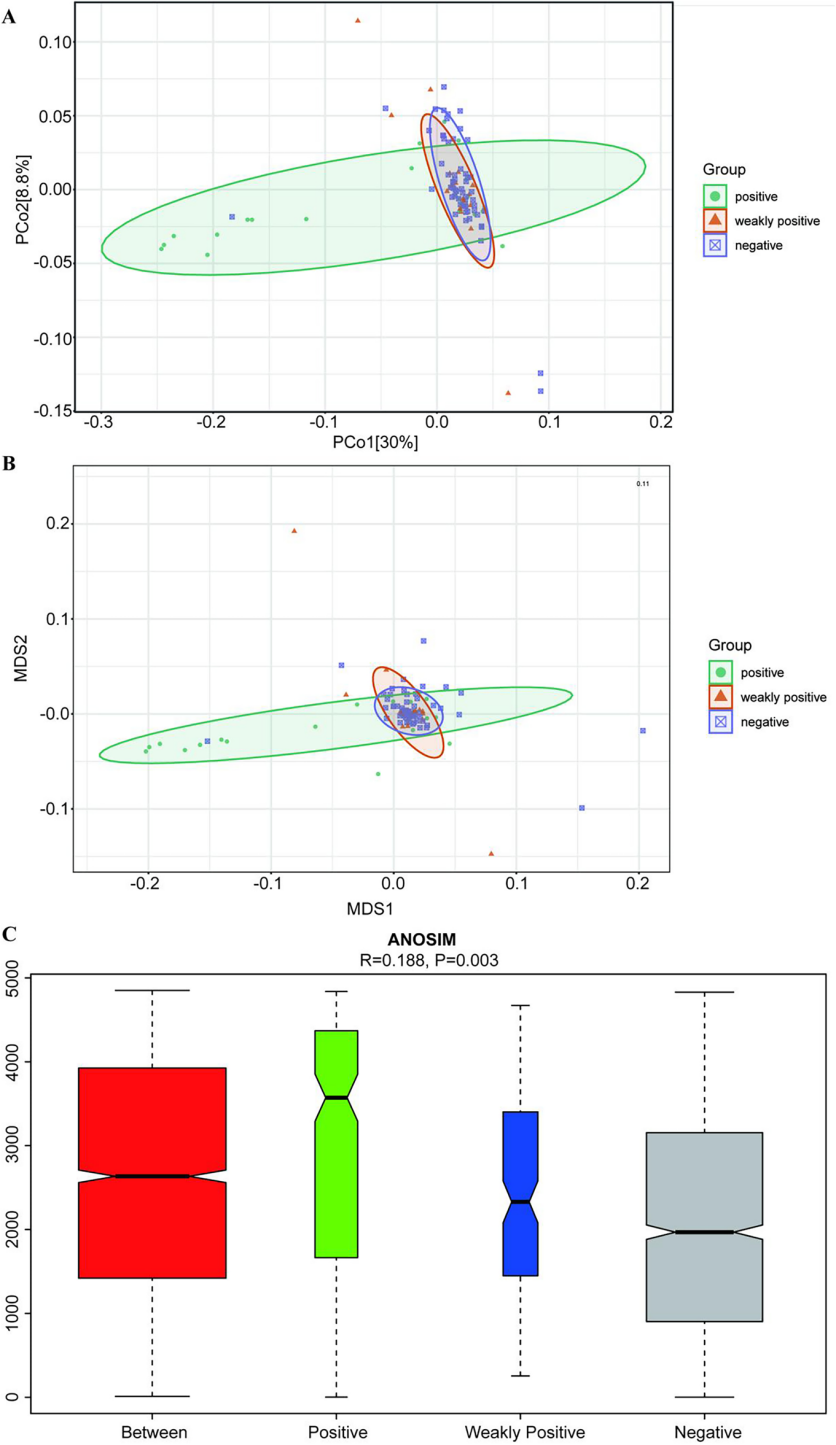
Differences in β-diversity index between the positive, negative, and weakly positive groups. (**A**) PCoA of weighted UniFrac showed a clear separation between the positive group and the weakly positive/negative group along the PCo1 axis, indicating a significant abnormality in the flora between the positive group and the remaining two groups. (**B**) Analysis of weighted UniFrac distances by NMDS showed a clear separation between the positive group and the weakly positive/negative group along the MDS1 axis, indicating a significant difference in flora between the positive group and the remaining two groups. (**C**) ANOSIM showed statistically significant differences between the groups (R = 0.188, *P* = 0.003). PCoA, principal-coordinate analysis; NMDS, non-metric multi-dimensional scaling.

The β-diversity was also analyzed to characterize the composition of microbial communities in samples by employing PCoA, NMDS, and Anosim analysis. Results of PCoA analysis revealed a clear separation between the positive and weakly positive/negative groups along the PCo1 axis of the endometrial microbiome (30%), with a large overlap between the weakly positive and negative groups with no significant difference (Fig. 4A). The NMDS plot showed a separated microbiome along the MDS1 axis between the positive and weakly positive/negative groups, indicating a significant difference between them; however, no significant difference was observed between the weakly positive and negative groups (Fig. 4B). Nevertheless, as shown in Fig. 4C, there was a statistically significant difference between the groups (R = 0.188, *P* = 0.003). These results collectively suggested a similarity between α- and β-diversity, implying an altered endometrial microbial community of endometritis patients.

### Comparison of microbiota in patients with endometritis and healthy controls

**Figure 5:**
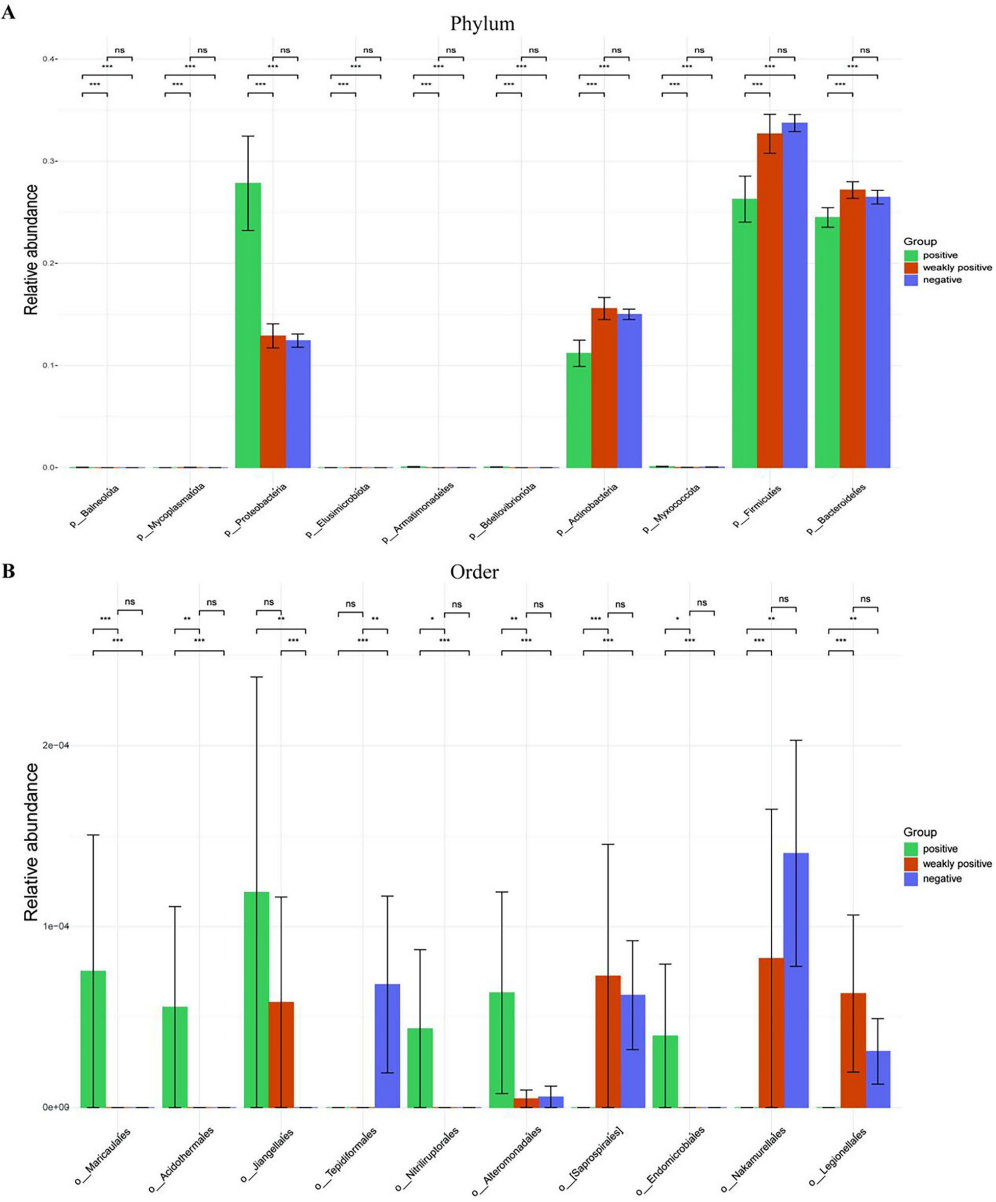
Differences in microbial abundance between the positive, weakly positive, and negative groups at the phylum/order level. (**A**) Differences of the top 10 microorganisms at the phylum level in the positive, weakly positive, and negative groups. (**B**) Differences of the top 10 microorganisms at the order level in the positive, weakly positive, and negative groups. ***P < 0.001. **P < 0.01. *P < 0.05. *ns* no significant.

The differential abundance test is an important part of analyzing microbial community data, which was conducted using MetaStat to analyze species abundance data across different groups and summarized the top 10 maps with large differences. Results showed a markedly elevated *Proteobacteria* abundance and reduced abundance of *Actinobacteria*, *Firmicutes*, and *Bacteroidetes* in the positive group at the phylum level, compared with the negative/weakly positive groups (Fig. 5A). Moreover, the abundance of *Maricaulales*, *Acidothermales*, *Jiangellales*, *Nitriliruptorales*, and *Endomicrobiales* was significantly higher, while that of *Saprospirales*, *Nakamurellales*, and *Legionellales* was markedly reduced in the positive group at the order level than that in the negative/weakly positive group (Fig. 5B). Nonetheless, the abundance of *Tepidiformales* was higher in the negative group, but no significant difference was observed between positive and weakly positive groups (Fig. 5B).

**Figure 6:**
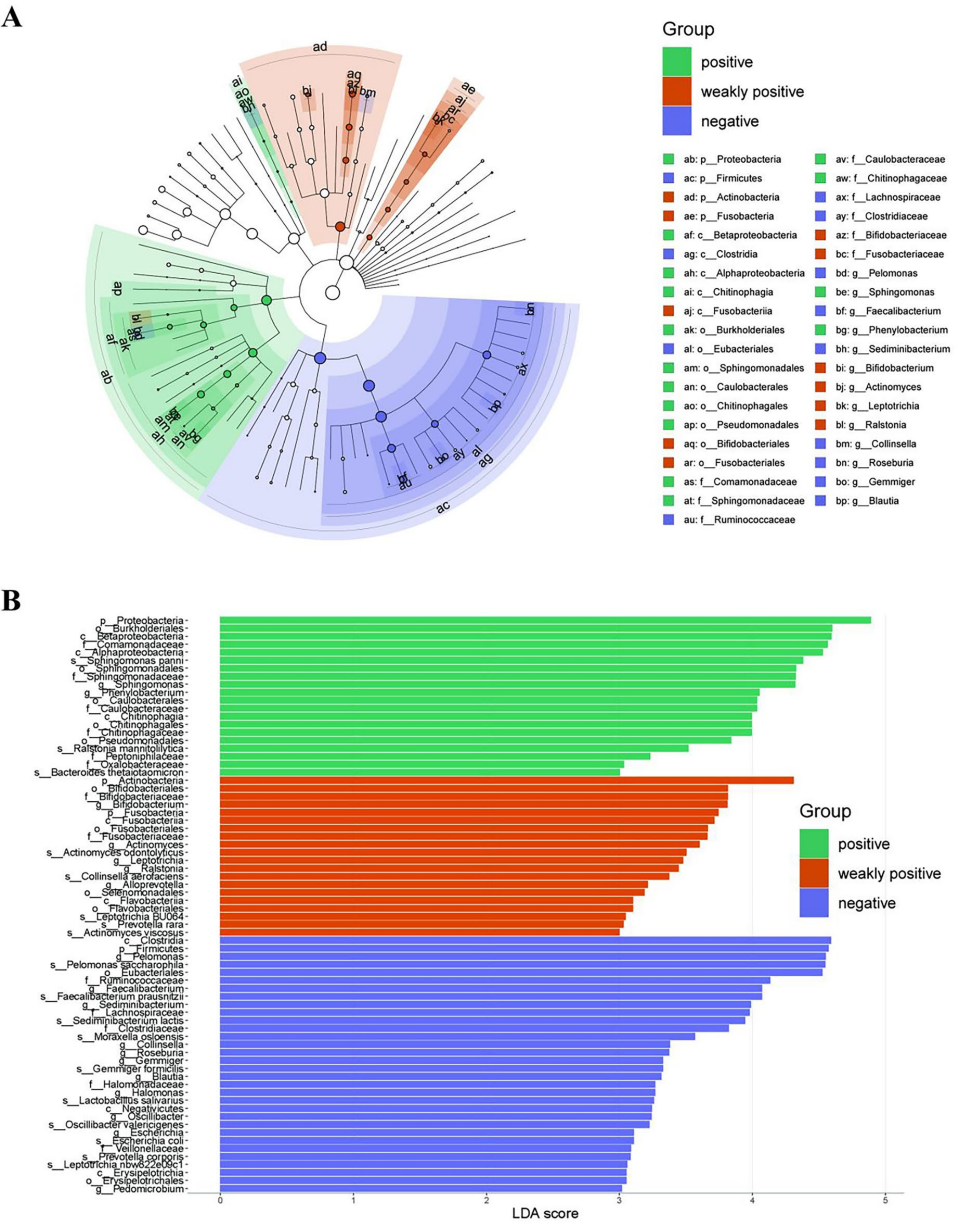
LEfSe analysis and LDA genus scores based on OTU characterizations of the microbiota in the positive, weakly positive, and negative groups. (**A**) The Cladogram was used to show the taxonomic hierarchy of the marker species in each group of samples. The different circles in the figure represent the seven taxonomic levels of phylum, order, family, and species, and each node represents a species classification at that level. The higher the abundance of the species the larger the node. (**B**) The threshold LDA score was set ≥ 3, and the longer lengths indicates more significant differences for that classification unit. *LEfSe* linear discriminant analysis effect size; LDA, linear discriminant analysis, *OTU* operational taxonomic unit.

It was then proceeded by comparing 72 species with significant abundance differences among all groups using LEfSe, an analytical tool for discovering and interpreting biomarkers (taxonomic units, pathways, genes) of high-latitude data. Moreover, microbial differences were evaluated using the Cladogram to exhibit the phylogenetic distribution of the endometrial microbiota. At the phylum level, *Proteobacteria* was dominant in the positive group; *Actinobacteria* and *Fusobacteria were* dominant in the weakly positive group; and Firmicutes was dominant in the negative group. At the class level, *Betaproteobacteria*, *Alphaproteobacteria*, and *Chitinophagia* were predominant in the positive group. In the other order, family, and genus levels, the microbes that dominated in each group were different (Fig. 6A). In addition, LDA genus scores were used to assess microbial differences. Results showed that 20 types of microbes, especially *Proteobacteria* (phylum level), *Burkholderiales* (order level), and *Betaproteobacteria* (class level), etc. were dominant in the positive group; 20 such as *Actinobacteria* (phylum level), *Bifidobacteriales* (order level), *Bifidobacteriaceae* (family level), etc. were dominant in the weakly positive group; while 32 comprising *Clostridia* (class level), *Firmicutes* (phylum level), *Pelomonas* (genus level), etc. were abundant in the negative group (Fig. 6B). All these results implied that the abundance of different types of microbiotas was changed in endometritis patients.

### Gene functional prediction

**Figure 7:**
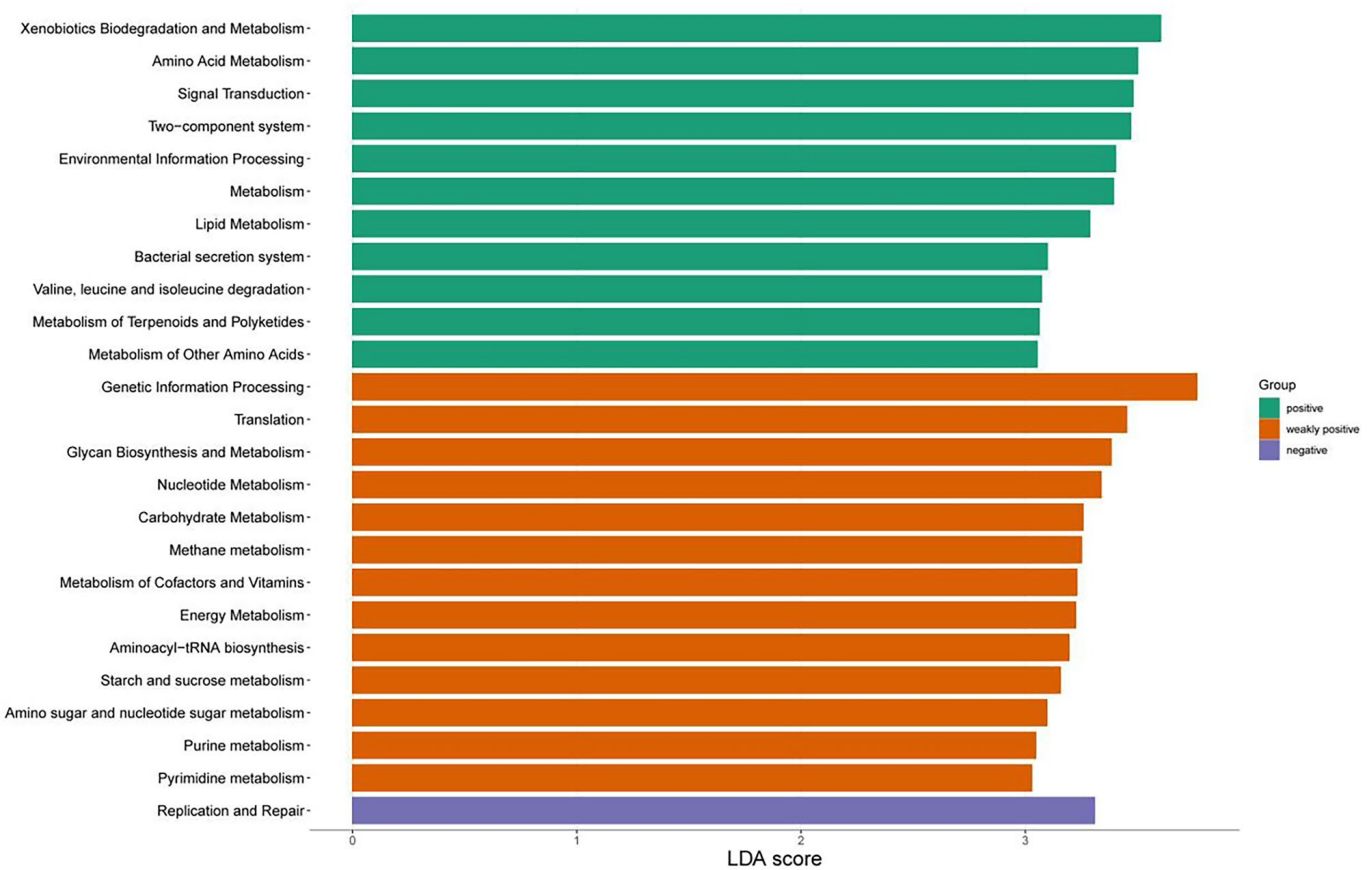
Functional predictive analysis. The KEGG pathways with significantly different gene functions between groups were screened. Threshold LDA score ≥ 3. The longer lengths indicated more significant differences for that classification unit. KEGG, Kyoto Encyclopedia of Genes and Genomes; LDA, linear discriminant analysis.

Functional and metabolic alterations in the endometrial microbiome were analyzed by extracting functional annotation data from whole prokaryotic genomes based on 16S rRNA sequencing in the KEGG database, which were then compared with this study's results. As shown in Figs. 7, 11 pathways, especially metabolism, were enriched in the positive group; 13 pathways, particularly genetic information processing, were enriched in the weakly positive group; however, only the replication and repair pathway was enriched in the negative group. As shown in Supplementary Fig. 2. Glycan biosynthesis and metabolism was enriched in the negative group, while two-component system, signal transduction, and environmental information processing pathways were enriched in the positive group.

### Disease risk prediction

**Figure 8:**
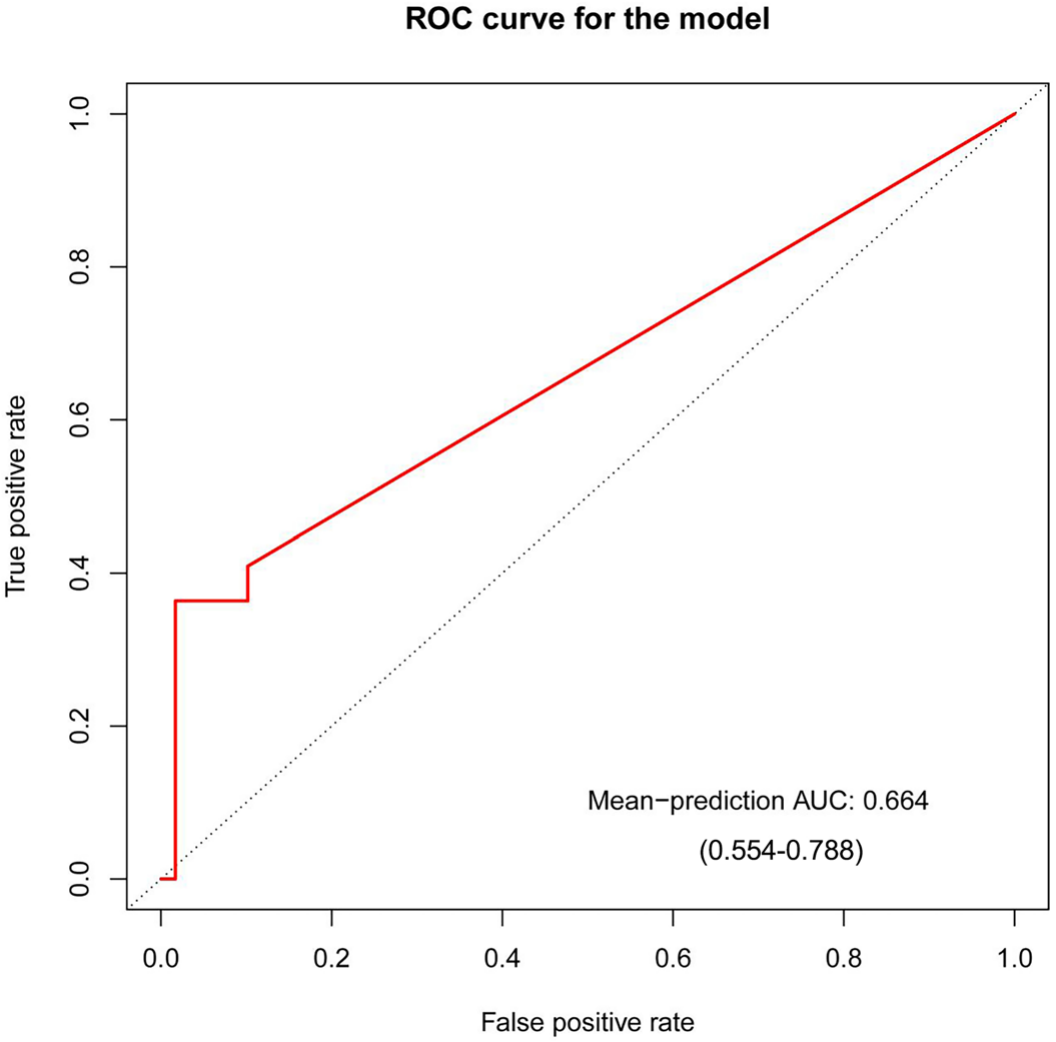
The diagnostic value of endometrial microorganisms for endometritis using a random forest model. The mean-prediction AUC of 0.664 from the ROC curve. The horizontal axis is false positive rate and the vertical axis is true positive rate. *AUC* area under curve, *ROC* receiver operating characteristic.

A mathematical model based on the sample data was developed and trained using random forest regression, while the diagnostic value of the endometrial microorganisms was evaluated using the ROC curve. Results revealed an area under the curve (AUC) value of 0.664 (95% CI 0.554–0.788; Fig. 8) for the developed model, cementing its potential diagnosed value. Hysteroscopy, HE staining and CD38 immunohistochemical staining are commonly used for CE diagnosis. The AUC value of hysteroscopy was 0.6412 (95% CI 0.5565–0.7259), and AUC of HE staining was 0.7361 (95% CI 0.6571–0.8150), and the AUC of CD38 immunohistochemistry was 0.5894 (95% CI 0.5342–0.6445) (Supplementary Fig. 3). The combination of microorganisms and HE staining had an AUC value of 0.859 (95% CI 0.7795–0.9384; Supplementary Fig. 4). The results suggested that the diagnosis performance of microbiota was lower than HE staining, but higher than hysteroscopy and CD38 immunohistochemistry. The combination of microbiota and HE can better diagnose endometritis.

### *C.t* alleviates inflammation response in mice caused by *S. aureus*

**Figure 9:**
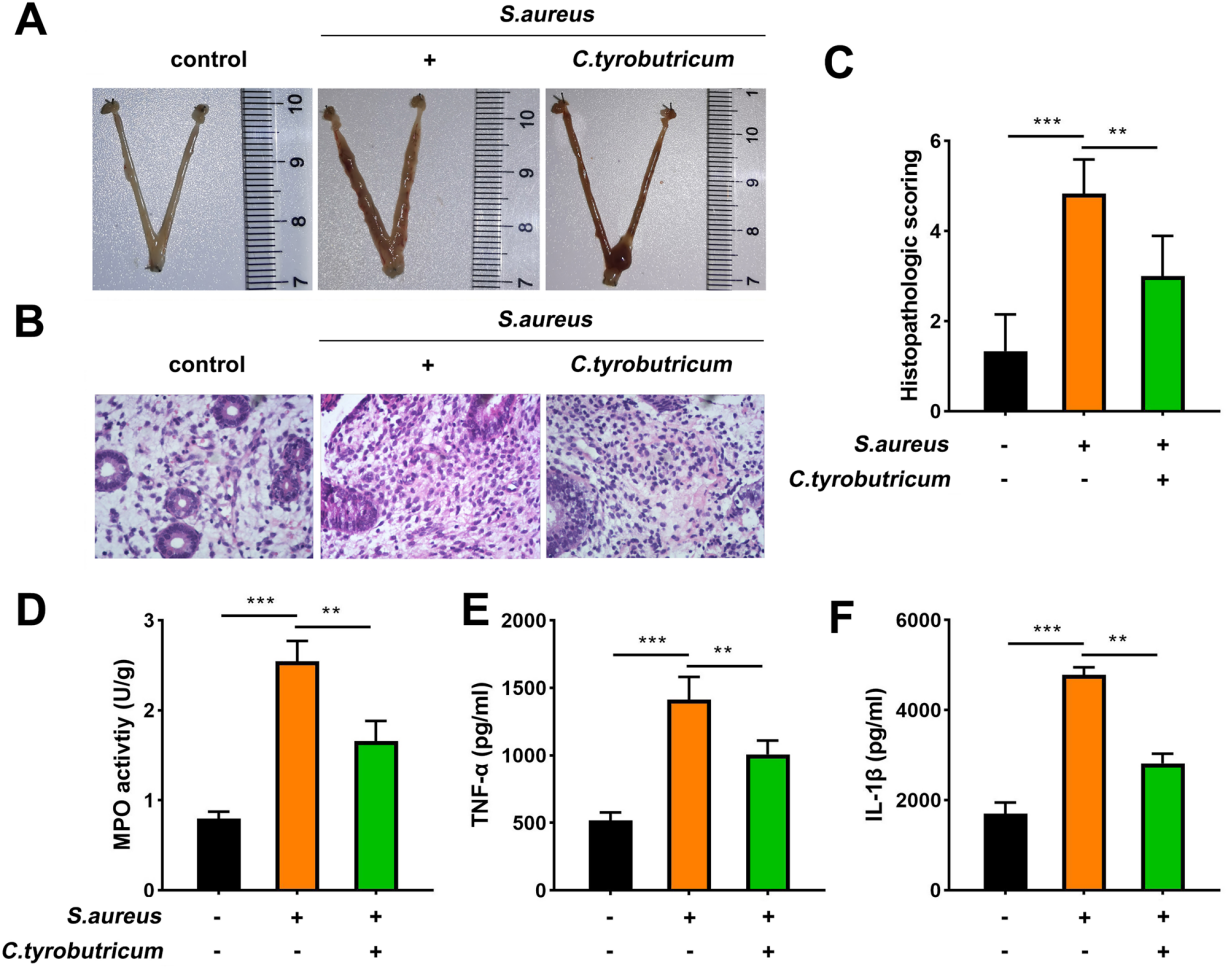
*C.t* alleviates inflammation response in mice caused by S. *aureus*. (**A**) Uterine was obtained from mice in each group and imaged. (**B**) Representative images of H&E staining showed the pathological changes in the endometrial tissues. (**C**) MPO activity in the endometrial tissues of mice in each group. (**D**) TNF-α and (**E**) IL-1β levels in the serum of mice in each group were measured using ELISA. H&E, hematoxylin and eosin; MPO, myeloperoxidase; ELISA, enzyme-linked immunosorbent assay. ***P < 0.001, **P < 0.01.

*S. aureus* was used to establish an endometritis mouse model, followed by administering *C.t* to investigate its role in vivo. For this purpose, mice's uterus was collected, and results showed that *S. aureus* induced an obvious erythema in the uterus of mice, which were reduced following *C.t* administration (Fig. 9A). Hematoxylin & eosin (H&E) staining results of endometrial samples showed that *S. aureus* resulted in abnormal pathological changes in the endometrium, including edema and inflammatory cell infiltration, which were alleviated following *C.t* administration (Fig. 9B,C). Similarly, an increased MPO activity in the endometrial tissues was observed in the *S. aureus*-treated mice, which was decreased following *C.t* administration (Fig. 9D). Analysis of mice serum TNF-α and IL-1β levels revealed their elevated levels post-*S. aureus* treatment, which was counteracted partly by *C.t* (Fig. 9D,E). All these results collectively suggested that *S. aureus* treatment translated into the occurrence of endometritis, which was attenuated with *C.t* treatment.

### *C.t* inhibits endometrial epithelial barrier damage caused by *S. aureus*

**Figure 10:**
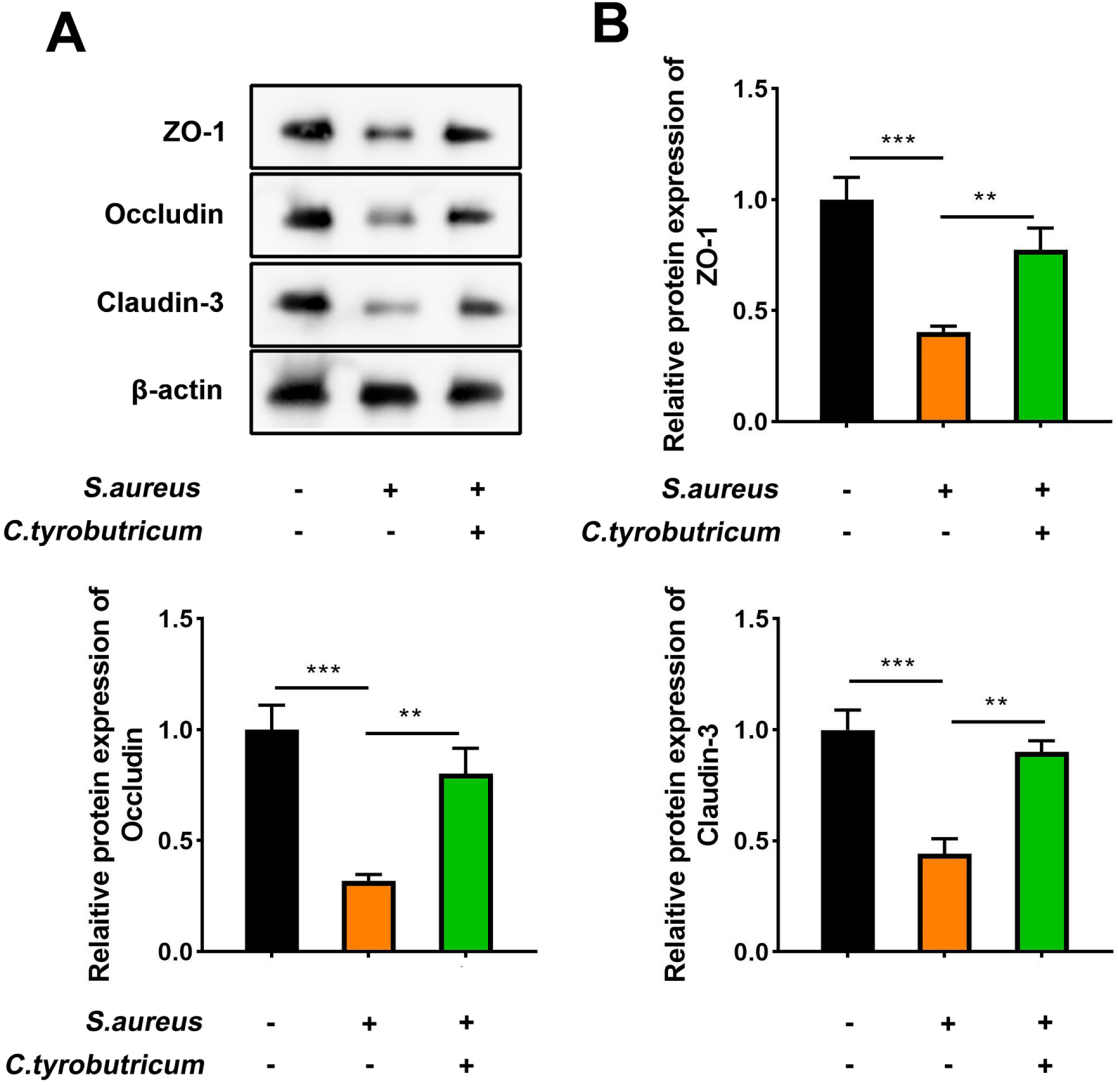
*C.t* inhibits endometrial epithelial barrier damage caused by S. *aureus*. (**A**) Represented protein bands of ZO-1, occludin, and claudin-3. β-actin was the internal control. (**B**) Protein levels of ZO-1, occludin, and claudin-3 were quantified. Original blots are presented in Supplementary Fig. 1. The samples derive from the same experiment and that gels/blots were processed in parallel. ***P < 0.001, **P < 0.01.

The intact endometrial epithelial barrier prevents exogenous irritation and bacterial infection, and the compromised epithelial barrier promotes the spread of infection^25^. Hence, we measured the levels of several epithelial barrier markers via western blotting, and the results revealed that the levels of ZO-1, occludin, and claudin-3 were decreased post-*S. aureus* treatment, which was reversed with *C.t* intervention (Fig. 10A,B). These results collectively suggested that *S. aureus* was involved in damaging the endometrial epithelial barrier in mice, which was attenuated with *C.t* administration.

### *C.t* suppresses the activation of the TLR4/NF-κB pathway induced by *S. aureus*

**Figure 11:**
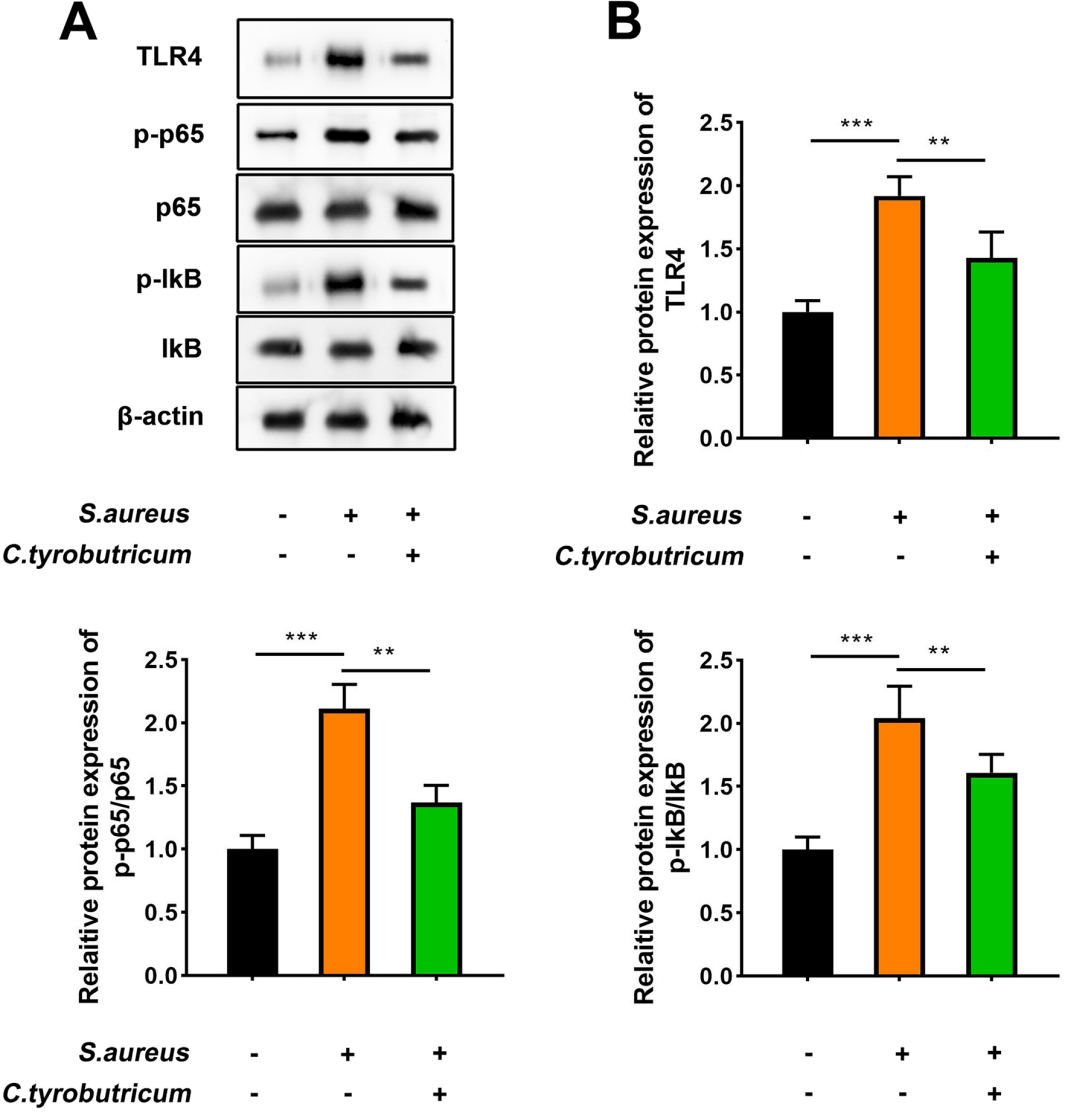
*C.t* suppresses the activation of the TLR4/NF-κB pathway induced by S. aureus. (**A**) Represented protein bands of TLR4, p-p65, p65, p-IKB, and IKB. β-actin was the internal control. (**B**) Protein levels of TLR4, p-p65/p65, and p-IKB/IKB were quantified. Original blots are presented in Supplementary Fig. 1. The samples derive from the same experiment and that gels/blots were processed in parallel. ***P < 0.001, **P < 0.01.

The TLR4/NF-κB pathway is a recognized inflammatory signaling pathway associated with the inflammatory response in endometritis^26^. Hence, the levels of various factors involved in this pathway were quantified using western blotting, and results showed that *S. aureus* treatment increased the protein levels of TLR4, p-p65/p65, and p-IKB/IKB in the endometrial tissues of mice, which were reduced with *C.t* treatment (Fig. 11A,B), demonstrating that *S. aureus* treatment activated the TLR4/NF-κB pathway, which was inhibited with *C.t* treatment.

## Discussion

Chronic endometritis is an infection of the endometrium caused by pathogenic bacteria and persistent inflammatory cells dominated by lymphocytes and can often go unnoticed because of the lack of noticeable or mild clinical symptoms^27^. Nevertheless, a significant association between chronic endometritis and infertility has already been reported^28^. Past studies on endometritis have primarily focused on treatment methods and clinical investigations related to the likelihood of miscarriage after embryo implantation^29–31^, and limited attention has been paid to investigating endometritis pathology, particularly the alterations in the microbiome. A changed uterine microbiome in women with endometritis has already been reported, leading to an increased endometritis incidence^32^, thus highlighting an association between modifications in the intrauterine microbiome and the occurrence of endometritis. Therefore, in our study, we aimed to elucidate the alterations of microbiota in patients with endometritis in central China using 16S rRNA sequencing, which may provide a new theory for the pathogenesis and possible diagnostic markers of endometritis.

Most research on alterations in microbial communities in endometritis was primarily focused on animals^33,34^. Recently, changes in microbial community-induced diseases in women like vaginitis, abortion, pelvic inflammatory disease, postoperative infection, and endometritis have been reported^35^. Chen et al.^13^ have reported that the abundance of *Lactobacillus* is increased and those of *Pseudomonas* and *Cutibacterium* are increased in women with endometritis. Liang et al.^36^ have found that the abundance of microbiota is decreased in endometritis, with increased *Firmicutes* and decreased *Actinobacteriota*. We also measured microbiota abundance, and found that *Proteobacteria* and *Burkholderiales* were increased, while *Actinobacteriota* was decreased in patients with endometritis. These results are partly contrary to the results of the Liang et al.^36^ study, as their study results were that *Actinobacteriota* and *Proteobacteria* were decreased in endometritis. Moreover, we also found that the α-diversity of the microbiota was increased in positive group, which is consistent with Chen et al.^13^ study; however, Liang et al.^36^ considered that the α-diversity of the microbiota has no significant change in endometritis. According to the LEfSe results, a previous study showed that the abundance of *Phyllobacterium* and *Sphingomonas* is increased in patients with chronic endometritis^14^. Our study showed that *Sphingomonas* and *Phenylobacterium* abundance was increased in the positive control, suggesting the important role of *Sphingomonas* in endometritis.

The functions of microbiota were further analyzed. We found that 11 and 13 pathways are enriched in the positive and weakly positive groups, respectively, mainly metabolism. However, only one pathway was enriched in the negative group. Chen et al.^13^ have found that arginine and proline metabolism and retinol metabolism are enriched in endometritis. Another study showed that microbiotas in endometritis are enriched in the sucrose biosynthesis pathway^14^. Additionally, endometritis is associated with cysteine and methionine metabolism, arachidonic acid metabolism, and pyrimidine metabolism pathways in mice^37^. Similarly, we also found that endometritis was related to metabolism pathway in this study, but the results are not the same. The results showed that xenobiotics biodegradation and metabolism and amino acid metabolism were the top two enriched pathways in the positive group. The findings consistent with a previous study that amino acid metabolism in genital tract is associated with the susceptibility of chronic endometritis^38^. The negative group was only enriched in the replication and repair, which participates in immune response evasion in the context of endometritis^39^, suggesting this pathway contribute to improve endometritis. Metabolomics analysis may be needed to further elucidate the function of metabolites. Moreover, as compared with the negative group, two-component system, signal transduction, and environmental information processing pathway were increased, while glycan biosynthesis and metabolism pathway was increased in the negative group. The findings suggest that in addition to metabolism, the onset of endometritis is very complex, affected by a variety of factors. Differences in these pathways can be used to distinguish patients with endometritis from normal people, which may provide new ideas for the pathogenesis of endometritis.

The microbiota in endometritis holds significant potential as a biomarker, which was explored in this study via the ROC curve, where the AUC value was found to be 0.664, partly indicating the utilization of endometrial microorganisms for the diagnosing endometritis; Moreover, we compared the diagnostic performance of microbiota and existing diagnostic methods hysteroscopy, HE staining, and CD38 immunohistochemistry staining, and found that the diagnosis performance of microbiota was lower than HE staining, but higher than hysteroscopy and CD38 immunohistochemistry. Particularly, diagnosis using microbiota had poor sensitivity, suggesting a high rate of missed diagnosis based only on microorganisms. However, previous studies have reported a high efficacy of microbiota in disease diagnosis like the use of intestinal microbes for diagnosing epilepsy, where an AUC of 0.993 was reported^40^. Similarly, an AUC of 0.8325 was found for diagnosing autoimmune hepatitis using the microbiome^41^. Lower AUC values, in our case, might be related to the relatively closed environment of the uterus and the different stages of disease development. Our results showed insignificant differences between microbial communities among the positive and negative groups, suggesting that early endometritis does not significantly alter microbial composition and abundance. Moreover, compared to the gut microbiome, the endometrial microbiome was less active in terms of compositional changes. Therefore, we speculated that microbiological diagnosis may only be used as one of the auxiliary methods in clinical practice, and the combination of it with the current diagnostic methods may improve the diagnostic accuracy. We found that the combination of microbiota and HE with the highest AUC value alone had an AUC value of 0.859, which will improve the current difficult clinical diagnosis of endometritis.

The abundance of *Fusobacteria* at phylum and *Fusobacteriales* at order level was found to be reduced in the positive group, and based on the probiotic characteristics, *C.t* (*Fusobacteriales*) was selected for this study, and its impact on endometritis and the underlying molecular mechanism was investigated, in an *S. aureus*-induced endometritis animal model^23,42^. Previous studies have shown that *C.t* helps protect against colonic inflammation and inflammatory bowel disease^19,43^ and can also inhibit endometrial barrier damage and inflammation in endometritis mice^22^. Our results showed that *C.t* improved histopathology, alleviated inflammatory response, and inhibited endometrial epithelial barrier disruption, which was consistent with the previous study^22^. Molecular mechanism investigations of *C.t* action in endometritis revealed that the TLR4/NF-κB pathway plays a pivotal role in endometritis, e.g., catalpol has been found to inhibit inflammatory injury to the mouse uterus by suppressing the TLR4/NF-κB pathway activation^44^. Similarly, ISGylation has been reported to suppress inflammation in endometrial epithelial cells via TLR4/NF-κB pathway^45^. Moreover, *Clostridium butyricum* inactivates the NF-κB pathway to ameliorate *Escherichia coli*-induced endometritis^46^. However, whether *C.t* affected the TLR4/NF-κB pathway in endometritis remains unclear. Our results indicated that TLR4, p-p65/p65, and p-IKB/IKB levels were downregulated by *C.t* in *S. aureus*-induced endometritis mice, suggesting that *C.t* inactivates the *S. aureus*-induced TLR4/NF-κB pathway. In summary, *C.t* alleviates *S. aureus*-induced endometritis by inactivating the TLR4/NF-κB pathway. However, whether *C.t* alleviates endometritis by regulating other molecular mechanisms remains unknown. The dose, effect and pharmacokinetics of *C.t* in the clinical treatment of endometritis are important challenges to translate *C.t* into clinical application.

This study still has some limitations. Due to the small sample size used in this study, the geographical limitations of the sample population, and the unknown status of their antibiotic resistance, we obtained many different results from previous studies. Additionally, the cross-sectional design limits our understanding of microbial changes during the progression of endometritis. Besides, whether *C.t* can be used in the clinical treatment of endometritis is not sufficient evidence, and how *C.t* changes the altered microorganisms has not been studied.

## Conclusion

Microbial diversity and abundance are significantly altered in patients with endometritis with positive CD138 levels, but are not significantly different in weakly CD138-positive patients and patients with non-endometritis. The changes in microbiota have the potential to be the diagnostic value in endometritis. Moreover, *C.t* alleviates *S. aureus*-induced endometritis in mice by inactivating the TLR4/NF-κB pathway. This study will provide a strong theoretical basis for the diagnosis and treatment of endometritis.

### Supplementary Information

Supplementary Legends.

Supplementary Figure 1.

Supplementary Figure 2.

Supplementary Figure 3.

Supplementary Figure 4.

Supplementary Figure 5.

## Data Availability

Further information and requests for reagents should be directed to and will be fulfilled by Aiming Wang (one_army@163.com).
