# Supplementary material for: Microbiome dysbiosis in patients with chronic endometritis and Clostridium tyrobutyricum ameliorates chronic endometritis in mice
Source: Sci Rep. 2024 May 30;14:12455. doi: 10.1038/s41598-024-63382-4 (PMC11139922; doi:10.1038/s41598-024-63382-4)
Supplement: Supplementary file 1 — Supplementary Legends. [file 41598_2024_63382_MOESM1_ESM.docx]

**Supplementary Figure 1** The original protein bands.

**Supplementary Figure 2** Bar graph of enriched KEGG pathways in positive and negative groups. LDA score >3.

**Supplementary Figure 3** The diagnostic value of current diagnosis methods in endometritis, including hysteroscopy, HE staining, and CD38 immunohistochemistry staining.

**Supplementary Figure 4** The diagnostic value of microbiota combined with HE staining in endometritis.
