# Supplementary figures and images for: Microbiome dysbiosis in patients with chronic endometritis and Clostridium tyrobutyricum ameliorates chronic endometritis in mice
Source: Sci Rep. 2024 May 30;14:12455. doi: 10.1038/s41598-024-63382-4 (PMC11139922; doi:10.1038/s41598-024-63382-4)

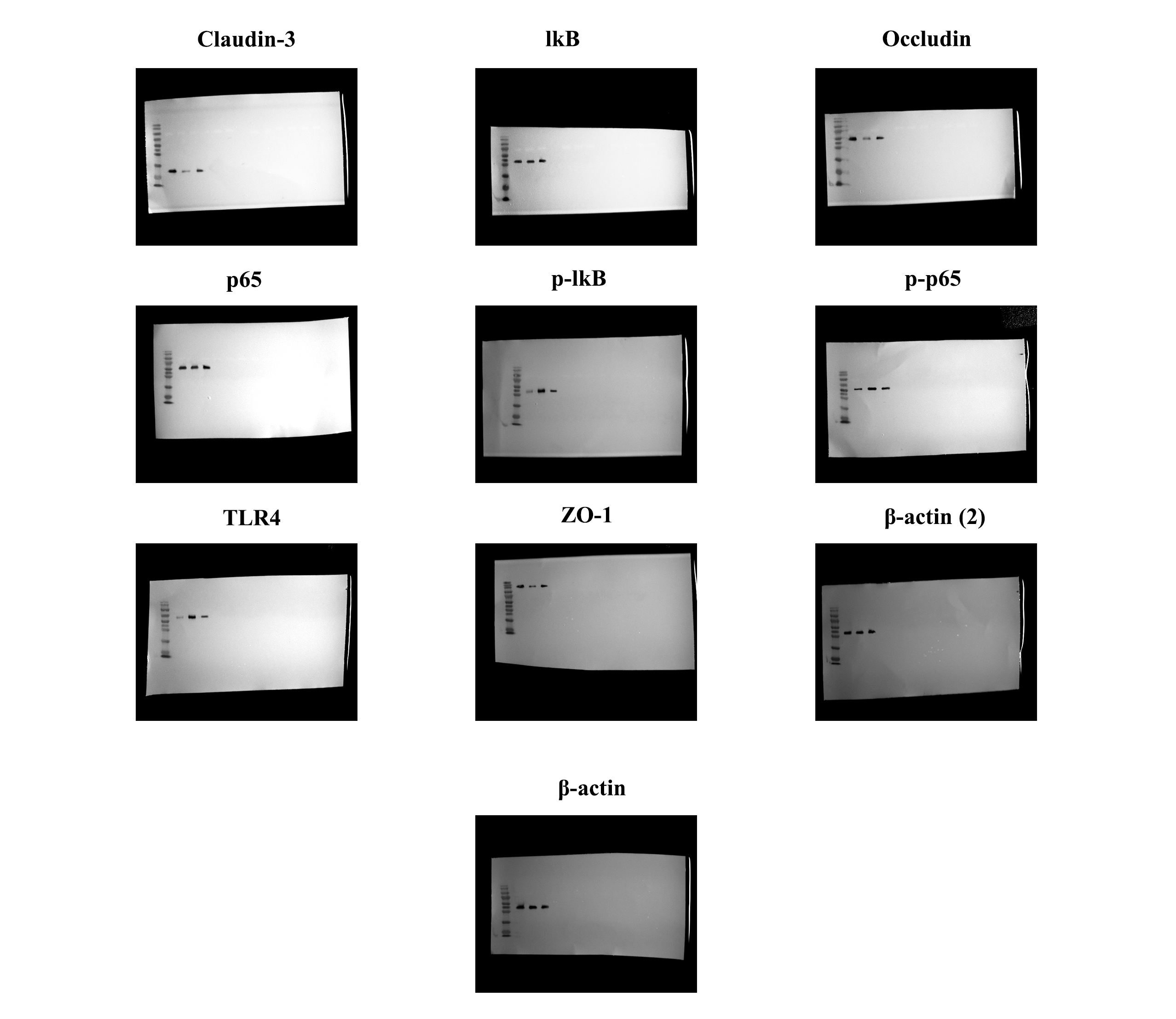

Supplement: Supplementary file 2 — Supplementary Figure 1. [file 41598_2024_63382_MOESM2_ESM.jpg]

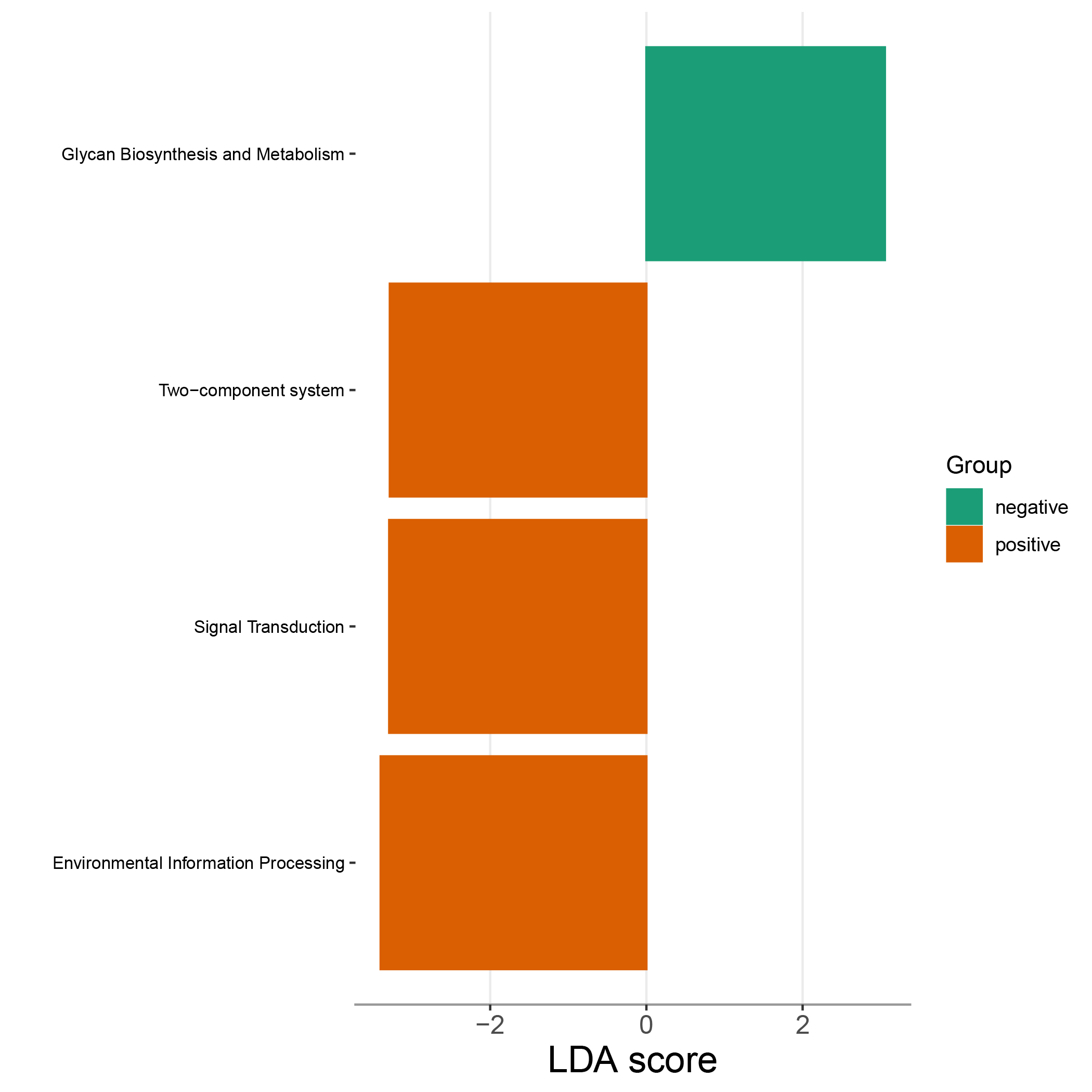

Supplement: Supplementary file 3 — Supplementary Figure 2. [file 41598_2024_63382_MOESM3_ESM.jpg]

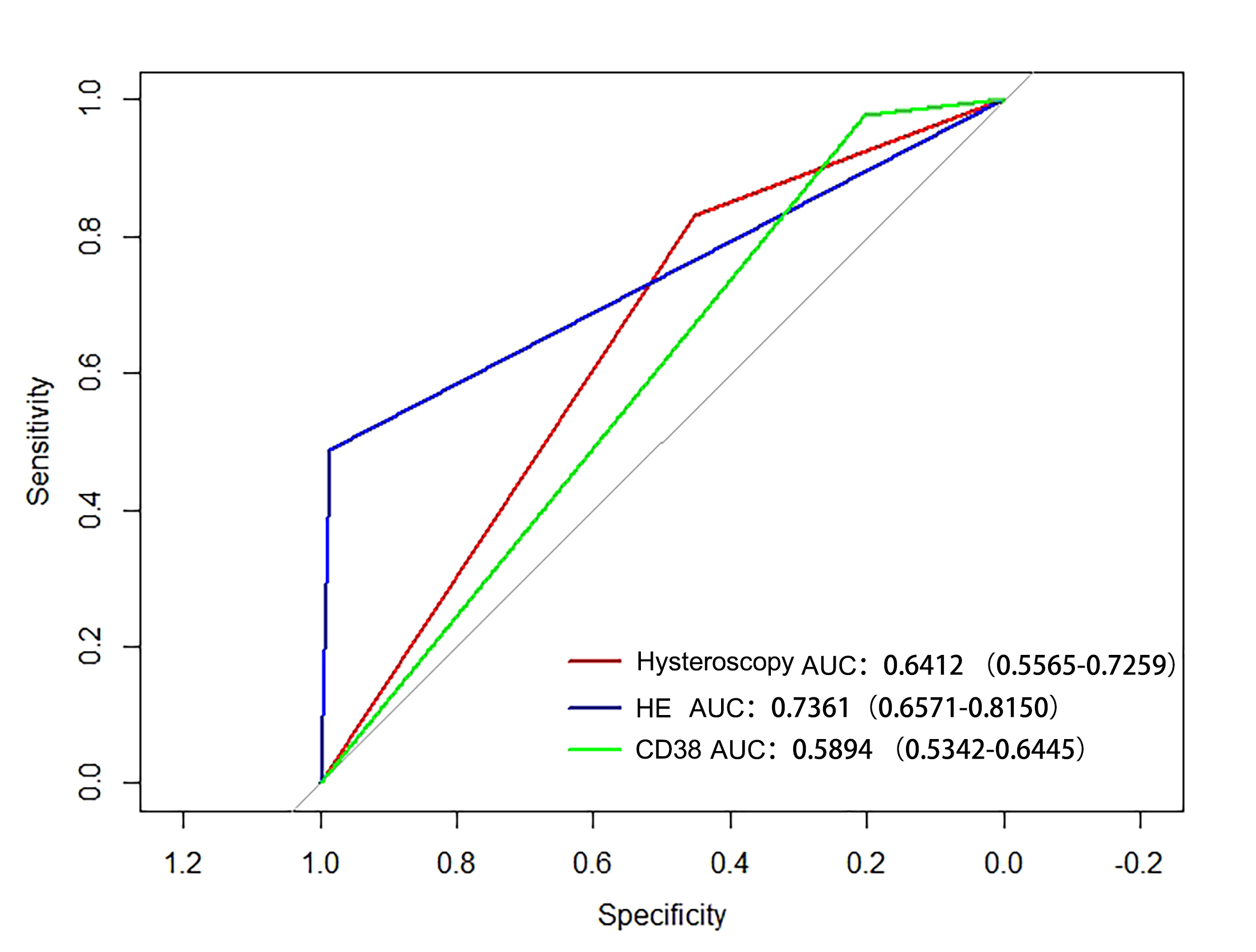

Supplement: Supplementary file 4 — Supplementary Figure 3. [file 41598_2024_63382_MOESM4_ESM.jpg]

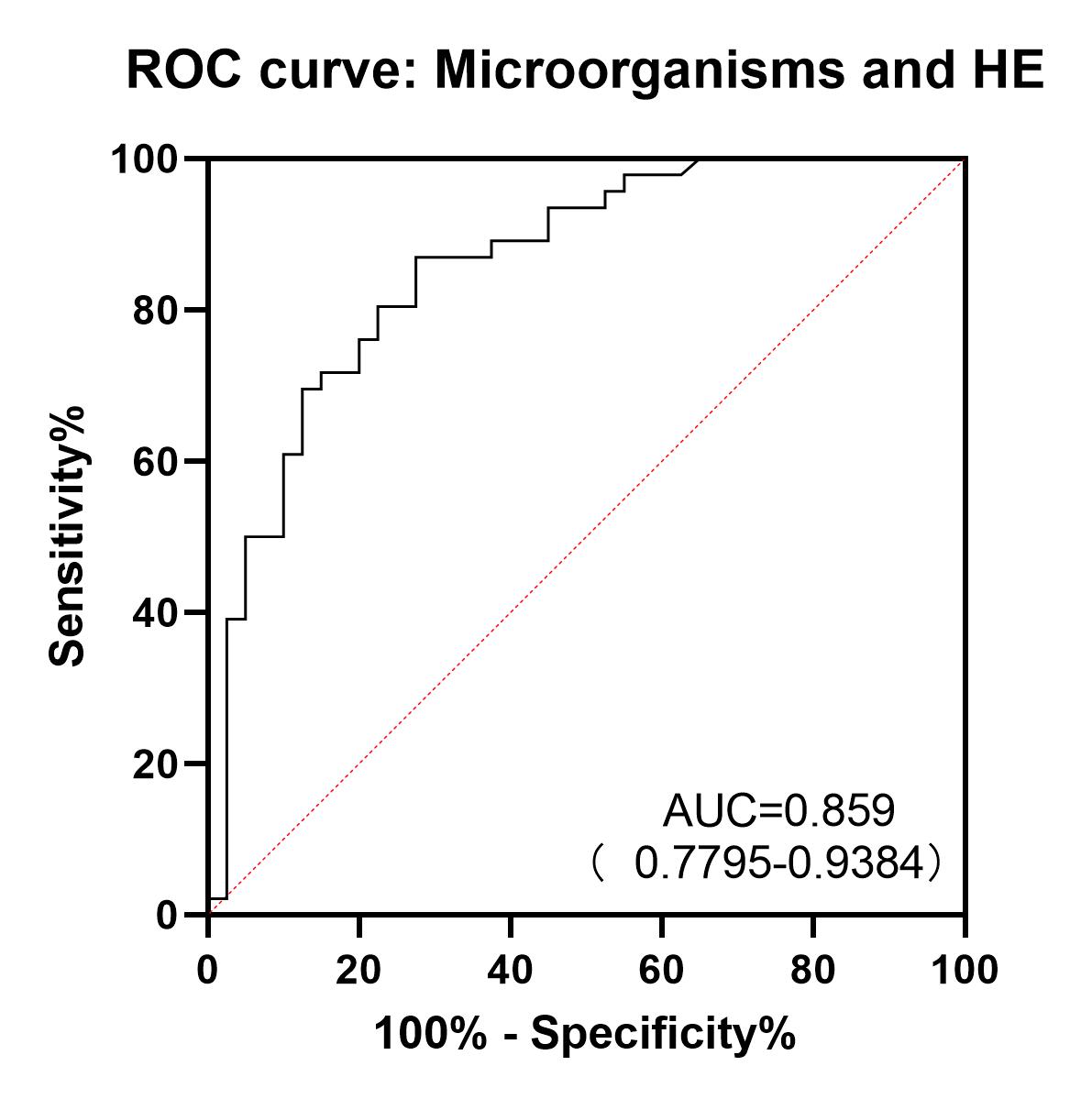

Supplement: Supplementary file 5 — Supplementary Figure 4. [file 41598_2024_63382_MOESM5_ESM.jpg]

|                                                                                     |                                                                                    |                                                                                     |
|-------------------------------------------------------------------------------------|------------------------------------------------------------------------------------|-------------------------------------------------------------------------------------|
| 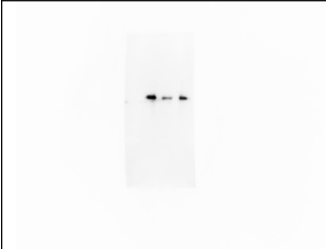   | 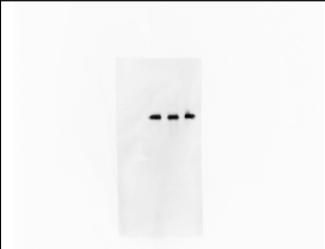  | 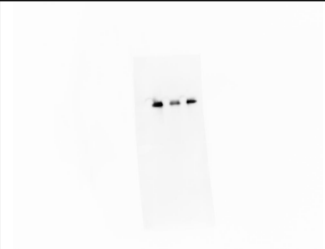  |
| Claudin-3                                                                           | IκB                                                                                | Occludin                                                                            |
| 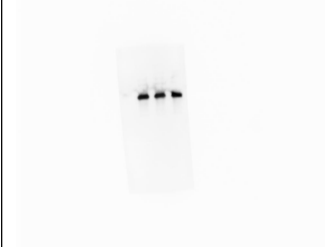   | 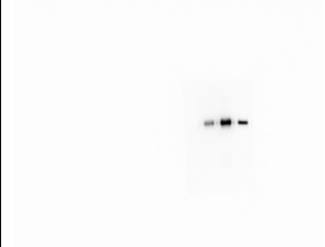  | 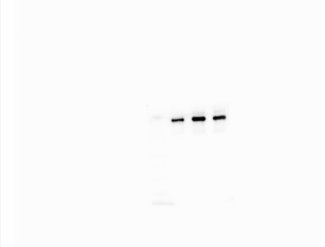  |
| p65                                                                                 | p-IκB                                                                              | p-p65                                                                               |
| 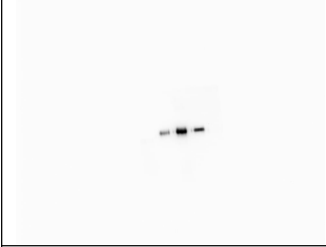  | 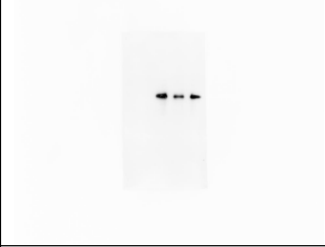 | 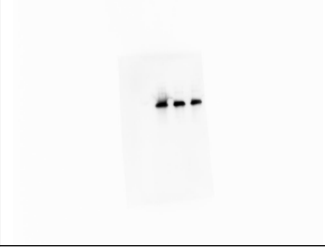 |
| TLR4                                                                                | ZO-1                                                                               | β-actin                                                                             |
| 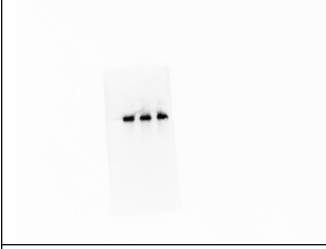 |                                                                                    |                                                                                     |
| β-axctin                                                                            |                                                                                    |                                                                                     |

Supplement: Supplementary file 6 — Supplementary Figure 5. [file 41598_2024_63382_MOESM6_ESM.pdf]
